# The Role of Stigma, Illness Acceptance, and Social Support in Mental Health Care Service Utilization Among Cancer Patients in Hungary

**DOI:** 10.1192/j.eurpsy.2025.1777

**Published:** 2025-08-26

**Authors:** R. Chowdhry, A. Kegye, E. Földesi, V. K. Bognár, G. Purebl, D. Őri

**Affiliations:** 1 Institute of Behavioral Sciences, Semmelweis University, Budapest, Hungary

## Abstract

**Introduction:**

An understanding of cancer from a psychological perspective is being increasingly prioritized in research and clinical care. Psychiatric disorders have been demonstrated to affect at least 35% and up to 55% of cancer patients throughout their trajectory. Further, cancer treatment adherence and health behaviors have been shown to have a marked decrease due to a series of mental health contributors. Therefore, comprehensive collaborative care interventions incorporating psychological care into oncological care has shown increased promise in reducing the burden of such comorbid diseases and promoting positive health outcomes. Social contexts, support, internal beliefs, and social perceptions significantly influence patients’ tendencies to utilize mental health services. An understanding of the psychosocial factors that influence mental health care utilization among cancer patients may promote greater utilization, evidence-based interventions, and consequently better health outcomes.

**Objectives:**

We aim to assess perceived stigma towards disease, perceived social support, and acceptance of the illness as related to rates of utilization among cancer patients undergoing treatment.

**Methods:**

We will measure the perceived stigma of the disease via the Hungarian version of the Stigma Scale for Chronic Illnesses (SSCI-8). The perceived social support will be measured by the Hungarian version of the Multidimensional Scale of Perceived Social Support (MSPSS) and the acceptance of illness will be measured via the Acceptance of Illness Scale (AIS) and Mini-Mental Adjustment to Cancer (Mini-MAC) to assess coping reactions to cancer. These surveys will be administered to 150 cancer patients at Semmelweis University in Budapest, Hungary and data collection will take place from November 2024 until February 2025.

**Results:**

This research is currently ongoing, and we will be able to present study results based on a sample of 150 individuals, demonstrating how perceived social support, perceived stigma of the disease, and illness acceptance relate to rates of the utilization of mental healthcare services such as psychotherapy and psychotropic medications, specifically among cancer patients in Hungary.

**Conclusions:**

An in-depth understanding of the social and psychological factors that influence positive health behaviors in the form of mental healthcare service utilization may inform the development of more tailored evidence-based collaborative care interventions.

**Disclosure of Interest:**

None Declared

